# Four Reasons to Question the Accuracy of a Biotic Index; the Risk of Metric Bias and the Scope to Improve Accuracy

**DOI:** 10.1371/journal.pone.0158383

**Published:** 2016-07-08

**Authors:** Kieran A. Monaghan

**Affiliations:** CESAM – Centre for Environmental and Marine Studies & Department of Biology, Universidade de Aveiro, Campus Universitario de Santiago, 3810-193, Aveiro, Portugal; University of Brighton, UNITED KINGDOM

## Abstract

Natural ecological variability and analytical design can bias the derived value of a biotic index through the variable influence of indicator body-size, abundance, richness, and ascribed tolerance scores. Descriptive statistics highlight this risk for 26 aquatic indicator systems; detailed analysis is provided for contrasting weighted-average indices applying the example of the BMWP, which has the best supporting data. Differences in body size between taxa from respective tolerance classes is a common feature of indicator systems; in some it represents a trend ranging from comparatively small pollution tolerant to larger intolerant organisms. Under this scenario, the propensity to collect a greater proportion of smaller organisms is associated with negative bias however, positive bias may occur when equipment (e.g. mesh-size) selectively samples larger organisms. Biotic indices are often derived from systems where indicator taxa are unevenly distributed along the gradient of tolerance classes. Such skews in indicator richness can distort index values in the direction of taxonomically rich indicator classes with the subsequent degree of bias related to the treatment of abundance data. The misclassification of indicator taxa causes bias that varies with the magnitude of the misclassification, the relative abundance of misclassified taxa and the treatment of abundance data. These artifacts of assessment design can compromise the ability to monitor biological quality. The statistical treatment of abundance data and the manipulation of indicator assignment and class richness can be used to improve index accuracy. While advances in methods of data collection (i.e. DNA barcoding) may facilitate improvement, the scope to reduce systematic bias is ultimately limited to a strategy of optimal compromise. The shortfall in accuracy must be addressed by statistical pragmatism. At any particular site, the net bias is a probabilistic function of the sample data, resulting in an error variance around an average deviation. Following standardized protocols and assigning precise reference conditions, the error variance of their comparative ratio (test-site:reference) can be measured and used to estimate the accuracy of the resultant assessment.

## Introduction

The unprecedented threats to earth’s ecosystems have given critical importance to the science of bioassessment [1, 2]. Progressive environmental laws, defined by biological criteria, offer a valuable opportunity to reduce biodiversity loss [3,4]. Attainment of their aims and objectives depends on the provision of accurate information about the ecosystems they are intended to protect. Obtaining a representative measurement of biological quality represents a considerable challenge [5]; over the last century a multitude of alternative approaches have been proposed [6]. The oldest and most widely employed is based on the assignment of indicator taxa and the subsequent interpretation of assemblage composition [7]. The concept of describing indicator assemblages in terms of a composite index was first applied to terrestrial plants [8]. It was subsequently embraced by freshwater scientists to measure the pollution status of plants and animals of freshwaters and, more recently, of estuarine and coastal waters [6, 9]. As the vanguard of bioassessment, biotic indices are fundamentally important to the management of biodiversity. Yet in sharp contrast to the scrutiny that the relatively simple (two-dimensional) indices of biodiversity have received [10], little effort has been made to gain a better understanding of how the component dimensions of biotic indices influence index performance.

**Fig 1:**
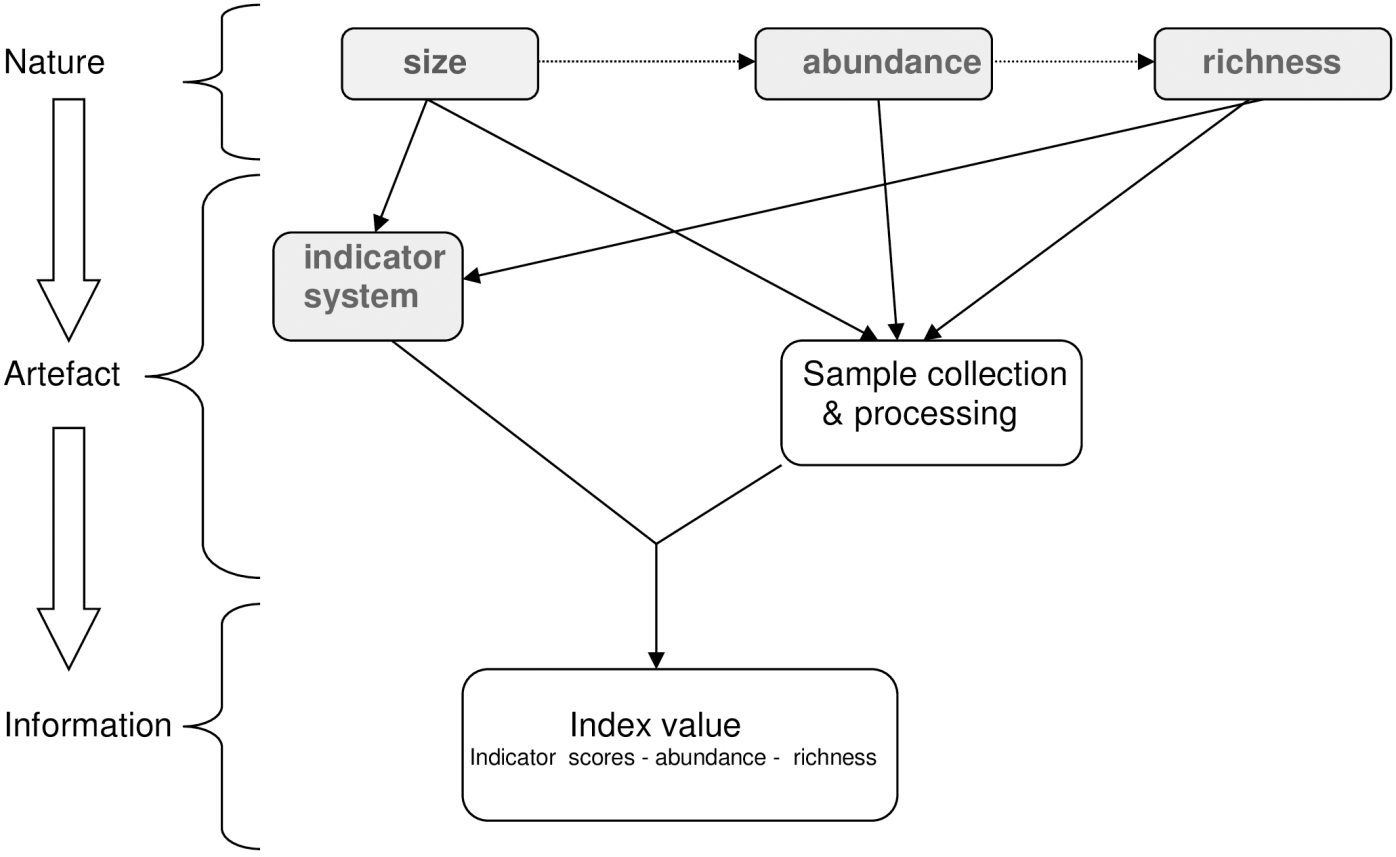
Interaction between the natural world and scientific strategy combine to define the derived value of a biotic index.

Knowledge of the natural world provides the starting point for a critique of ecological methods. In the case of the component dimensions of biotic indices, ecologists acknowledge a general relationship between richness and abundance [10, 11] and well-established patterns of abundance and body size [12]. Human perception and pragmatism are applied in describing abstract models of natural phenomena. In the case of biotic indices, the assignment of ranked indicator scores results in a contrived distribution of indicator richness (explicitly) and indicator size (implicitly) across the range of indicator classes. When samples are collected in the field and processed in the lab, the reality of the natural world is filtered according to the methods and equipment employed. The resultant “raw data” are ground-down once more as it is arranged in accordance with the indicator system and statistical algorithm(s) employed to generate the index value. During this analytical process, four parameters—body-size, abundance, richness and indicator score—contribute defining roles in the derived index value (Fig 1). Knowledge of their respective influence and potential synergistic/antagonistic interactions provides a theoretical perspective to review the risks of index bias.

Abundance is arguably the single most important parameter in ecology [13]; its treatment is fundamental to the myriad of published biotic indices [6,9]. While biomass may represent a more informative expression of abundance compared to count data, processing costs associated with data acquisition have precluded its widespread application [13,14]. The derivation of count data, has been guided by pragmatic trade-offs between precision, accuracy and processing costs. In the simplest scenario, presence/absence data, the abundance of organisms is neglected [15]. More commonly, indices are based on a count of all individuals [16,17,18]. Between these extremes various abundance-weighted treatments have been applied including the allocation of abundance categories [19], taxonomically defined abundance-weightings (based on presumed size-abundance relationships [20]), and the statistical transformation of count data [14].

The incorporation of abundance data can bias accuracy and reduce precision in two ways. Numerically dominant taxa can skew the result in the direction of their indicator scores. At the other extreme, presence/absence data or strongly transformed abundances can skew the result in favor of rare taxa by assigning them equal weighting as abundant taxa. These beguilingly simple alternatives need to be appreciated in context. Natural populations of species demonstrate differentially aggregated distributions [21, 22] with the degree of aggregation varying in time and space and in relation to the scale of the sampling unit [23]. Survey methods impose bias in capture efficiency. More generally, ecological communities are characterized by skewed distributions where the majority of species are rare and few are dominant [24]. Species may be rare for different reasons including vagrancy, implying that they are unrepresentative of the local environment [25]; this may be particularly problematic in aquatic habitats that wash-in allochtonous material and exacerbated when analysis is based on dead organisms (e.g. invertebrates, algae). Aquatic communities typically demonstrate inverse size-abundance relationships with abundance decreasing as size increases. Pollution is thought to distort size—abundance distributions, leading to scenarios where smaller organisms become proportionally more abundant in relation to larger organisms [26]. While indices of diversity aim to strike a pragmatic balance between the resultant patterns in richness and abundance [10], the classification of indicator taxa adds a further layer of complexity to biotic indices.

Factors affecting pollution tolerance and therefore indicator assignment are complex and can distort the precision of biotic indices. Organisms are differentially sensitive to different forms of environmental degradation, compromising the accuracy of generalized “pollution” indices [27]. Taxa can also differ in their sensitivity in time and space [28]. Yet the desire for greater regional integration has led to the application of indicator values over increasingly large geographic scales, resulting in highly generalized indicator values [29,30]. Pollution may interact with local environmental conditions, influencing the delivery and uptake of pollutants, exacerbating or ameliorating an individual’s susceptibility [31]. As organisms are ascribed tolerance ranks, human subjectivity can contribute to error; tolerance ranks are sometimes misclassified [32]. On a pragmatic level, classification of indicators at higher taxonomic levels (e.g. family) can represent a strategic compromise based on the average rank of constituent species [16] or, in a precautionary approach, the most tolerant species [33]. When the realized tolerance of an organism at a particular site differs from its classified tolerance value it will bias the derived index.

In bioassessment the overall measurement of error is based on the combined effect of multiple factors [34]. As contrasting biases may be counter-balanced, this holistic description of error can provide a useful “fit-for-purpose” evaluation with an interpreted meaning defined by the particular study. However, such case-specific knowledge limits an understanding of the respective causes of error and reduces the scope to evolve methods that might best address the emerging issues of global change. As biotic indices are multi-dimensional measurements, the variability of natural communities can confound the elucidation of the source(s) of measurement bias. To overcome this limitation, this study combines the analysis of real and idealized indicator systems and datasets to assess how indicator assignment, abundance, richness and body size impose fundamental limits on the range and accuracy of biotic indices. Explicitly, comparative analysis considers:

1. The skewed distribution of taxa across indicator ranks; when taxon richness of respective indicator classes differs.
2. Trends in organism size and pollution tolerance; when smaller organisms tend to be tolerant and larger organisms tend to be sensitive.
3. Misclassified taxa; when taxon occurrence reflects its’ true tolerance score but the indicator contributes an inaccurate score to the derived index.
4. How the treatment of abundance data influences the derived index value in the above scenarios.

## Methods

A wide range of biotic indicator systems were subject to descriptive review (Table 1).

**Table 1:** Indicator systems used to derive a biotic index for the bioassessment of inland and coastal waters. Indicator richness is the number of indicators in a discrete tolerance class; Null group refers to a non-linear range of indicator scores (i.e. an “empty group”); Evenness is Simpson’s D measuring indicator distribution across classes, D for upper/lower is based on the four indicator classes at the max/min of the indicator range.

	Acronym* (reference)	Stressor type	Indicator range	Indicator richness min-max	Null group	Evenness of indicator classes (D)	D for upper/ lower	reference
FRESHWATER MACROINVERTEBRATES	BMWP	Organic/ General	1–10	1–24	Yes	0.8094	0.555 0.507	Armitage et al. 1983
	IBMWP	Organic/ General	1–10	2–27	Yes	0.8468	0.584 0.592	Alba-Tercedor & Pujante 2000
	FBI	Organic/ General	0–10	2–17	No	0.871	0.740 0.748	Hilsenhoff 1987
	BI	Organic/ General	0–10	13–56	No	0.893	0.713 0.744	Hilsenhoff 1988
	AWIC	Acidity	1–6	3–33	Yes	0.524	0.373 0.712	Davy-Bowker et al. 2005
	LIFE	Flow	1–6	3–38	Yes	0.651	0.578 0.623	Extence et al. 1998
	SIGNAL2-fam	General	1–10	8–28	No	0.883	0.721 0.747	Chessman 2003
	SIGNAL2-order	General		1–8	Yes	0.8466	0.640 0.737	Chessman 2003
	MCI	Organic	1–10	3–34	No	0.854	0.708 0.545	Stark & Maxted 2007
	panUS_*nutrient*_	Nutrients	1–10	9–11	No	0.900	0.749 0.750	Carlisle et al. 2007
	panUS_*DO/temp*_	DO/ Temp.	1–10	9–11	No	0.900	0.750 0.750	Carlisle et al. 2007
FRESHWATER DIATOMS	TDI	Trophic N+P	1–5	196–585	No	0.762	0.710 0.693	Kelly 1998
	Trophic VD	Trophic	1–7	17–188	No	0.799	0.708 0.598	van Dam et al. 1994
	CEE	General	1–12	14–148	No	0.863	0.722 0.739	Descy & Coste 1991
	IPS	General	1–3	1–947	Yes	0.789	0.201 0.130	Cemagref 1982
	Saprobic-W	Organic	2–3	15–162	No	0.155	NA	Wanatabe et al. 1990
	Saprobic-S	Organic	0.1–4.0	1–79	Yes	0.949	0.736 0.524	Sládeček, 1986
	Halobion	Salts	1–4	3–112	No	0.562	NA	Ziemann (1971)
	pH VD	Acidity	1–6	1–310	No	0.702	0.650 0.581	van Dam et al. 1994
	Descy	Organic	1–5	4–54	No	0.647	0.611 0.620	Descy 1979
	Pan-Euro trophic	Nutrients	1–10* intermeds	1–71	Yes (3)	0.709	0.578 0.326	Besse-Lototskaya et al. 2011
MARINE BENTHOS	BQI west	Organic	1–15	1–56	Yes	0.901	0.686 0.563	Leonardsson et al. 2009
	BQIeast	Organic	1–15	4–32	Yes	0.682	NA	Leonardsson et al. 2009
	BITS	General	1–3	22–285	No	0.456	NA	Mistri & Munari 2008
	ABMI	General	1–5	12–474	No	0.587	0.587 0.575	Borja et al. 2000
	BENTIX	General	2–3	31–81	No	0.400	NA	Simboura & Zenetos 2002

*for convenience (not necessarily official acronym), see citation for definitive reference (S1 File).

Detailed analysis was based on the seminal example of the BMWP [15]. It was selected to exemplify biotic indices in general because of its widespread influence [35, 36, 37] and the wealth of supplementary information on its constituent taxa [32,33, 38, 39, 40]. Derivation of the biotic index value is based on contrasting treatments of abundance data (see below). The comparable risk of bias for biotic indices based on alternative indicator systems can be inferred from their respective summary statistics (Table 1).

The BMWP system incorporates 85 taxa (defined by family, except Oligochaeta), respectively ascribed to indicator rank scores ranging from 1–10, that correspond to a perceived quality gradient from pollution tolerant (one) to intolerant (ten). No indicators are ascribed the rank score nine, which acts as a null (empty) group. Assessment of the statistical characteristics of the BMWP was facilitated by comparison with a hypothetical indicator system (I_H_), represented by 100 indicators with 10 taxa ascribed to each of the ten indicator classes. Index values were derived according to a weighted-average of respective indicator abundance, where abundance was based on a range of increasingly severe transformations: raw abundance, square-root, logarithmic, presence/absence.

$$Index\;value\;\left(abundance\;weighted-average\right)=\;\frac{\sum a_{j}s_{j}}{\sum a_{j}}$$

where: *a*_*j*_ = relative abundance of species j; *s*_*j*_ = pollution tolerance score

Numerous researchers have proposed that the indicator mode provides a more accurate estimate of environmental conditions than a derived weighted-average [41]. The counter argument is that the mode discards information that is integrated within a weighted-average. As indicator analysis assumes species are distributed in relation to their environmental optima [8], theory suggests that under ideal conditions the derived weighted-average and the mode will coincide. While deviation from this theoretical scenario can arise from competitive displacement [42], it can also result from the intrinsic properties of index design and survey protocols. As this review is focused on the latter, comparative analysis is based on the assumption that the “true” index value corresponds to the indicator mode; herein a deviation from the mode is considered to represent bias.

**Fig 2:**
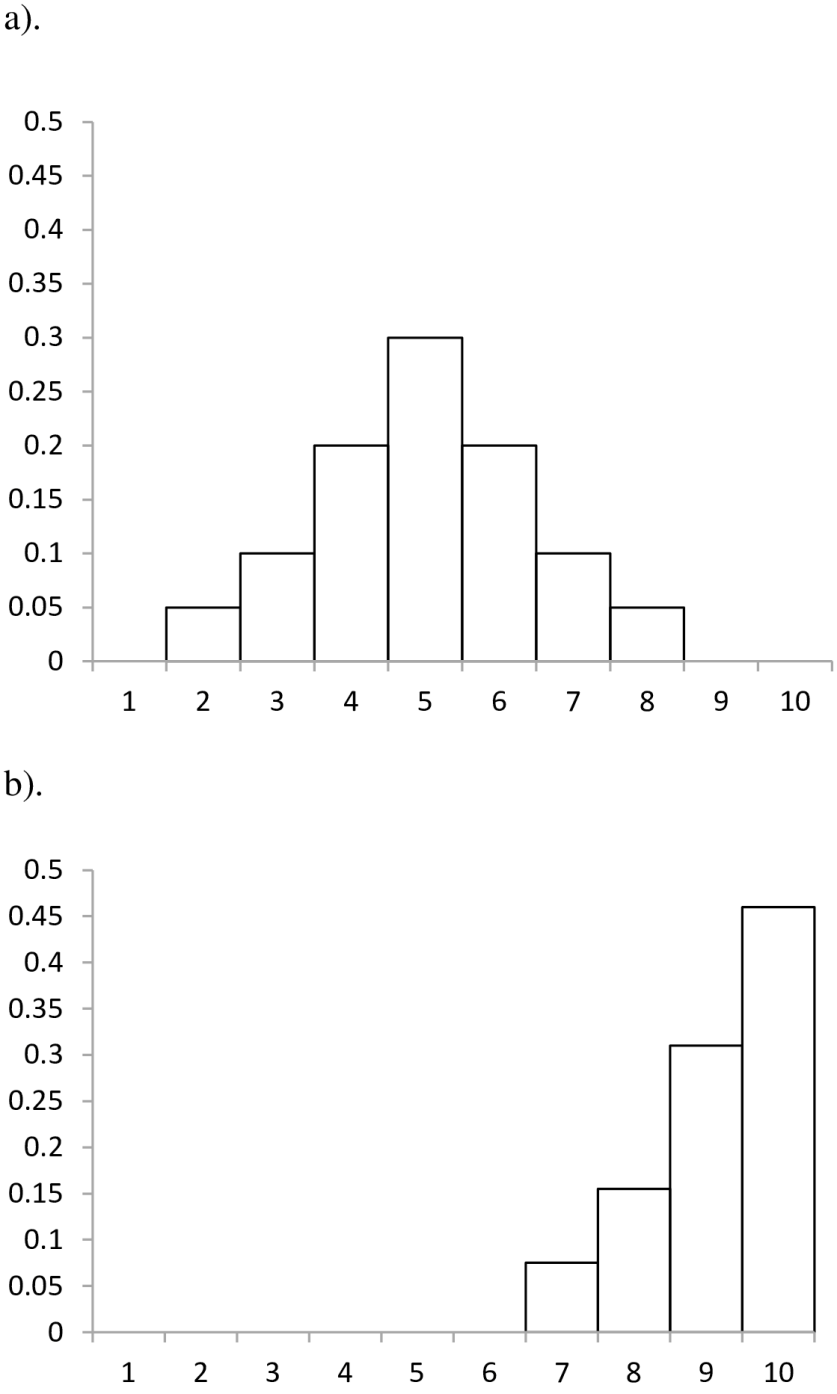
The unimodal probability distribution applied to indicator selection in simulation models illustrating two generalized examples. a). A symmetric distribution ranging across 7 indicator classes that occurs for mid-range scores defined by probabilities = 0.05,0.10,0.20,0.30,0.2,0.1,0.05. b). A truncated distribution that occurs for end-group scores, in this case associated with a mode of 10 where the “missing” probabilities, totaling 0.35 (i.e., the right-hand probabilities = 0.20,0.10,0.05, respectively corresponding to the non-defined indicator ranks of 11,12,13) are divided in proportion of the indicator ranks present (giving truncation-adjusted probabilities = 0.8,0.15,0.31,0.46).

All statistical analyses were carried out in R [43]. Simulation models were based on 20 replicates, each sampling 3000 individuals. Simulations were defined by a unimodal response function that spanned a fixed range of indicator classes. For mid-range modal values (indicator ranks 4–7), the response function was symmetric and spanned 7 rank scores (70% of the range; Fig 2a). For modal values at the extremes of the indicator range (1–3 and 8–10) the response function was truncated (as there are no indicator ranks <1 or >10). Under these scenarios the “lost” proportion of the symmetric distribution—that would be assigned to the absent indicator ranks (i.e. hypothetical indicators <1 or >10)–were redistributed in proportion amongst the indicator classes present (Fig 2b). Within rank classes all taxa had an equal probability of selection.

### Indicator evenness

Simpson’s diversity [44] was used to summarize the evenness of taxa across indicator classes. Evenness considered the entire range of indicator scores and, additionally, the lower and upper limits (i.e., the evenness of the four sequential indicator classes representing the respectively highest and lowest indicator ranks).

The effects of skewed indicator richness were elucidated by simulating specific scenarios of an increasing skew in the richness of the modal class and a single adjacent class. The initial even distribution of I_H_ (10 indicators per class) was progressively skewed by transferring modal taxa to the designated adjacent class. The influence of distance between the skewed classes was assessed by locating the enriched class 1, 2 and 3 ranks from the mode. Sampling was based on a symmetric, unimodal distribution (Fig 2a). For the BMWP system, skewed richness was assessed by comparing the index values from a series of simulations on the BMWP and the idealized system, I_H_, where the mode ranged from 1 to 10.

### Size bias

Ecological theory predicts a relationship between body-size and disturbance that has been extended to incorporate pollution, whereby smaller organisms are regarded as more pollution tolerant than larger organisms [26]. The consequence of a size-tolerance bias was investigated by defining an extreme size-biased indicator system (I_Hs_) where organism size and indicator scores were linearly correlated and associated with consequent differences in indicator densities (Table 2). Body-size—indicator interactions were assessed by considering a hypothetical habitat where space (n = 3000) could be occupied by one or more individual, depending on organism size. Habitat space was defined in terms of quality niches, corresponding to indicator scores, assigned in direct proportion to the unimodal response function applied in sample collection (as above). Overall size bias was assessed by comparing index values from simulations where the mode ranged from 1 to 10 for I_Hs_ vs the non-size-biased indicator system, I_H_. Specific size-bias issues considered the decimation (reduction by 90%) of the largest taxa (to mimic selective predation, habitat loss, etc.), where the resultant vacant space was colonized by indicators (drawn from the range of quality classes present) that were assigned (i) randomly, (ii) with a probability inversely proportional to organism size (i.e. smaller taxa had a higher probability of colonization). Finally, scenarios where the smallest, lowest scoring taxa were beyond the limits of detection were simulated (to mimic the effects of increasing mesh-size).

**Table 2:** Hypothetical size-biased indicator system (I_Hs_) where macroinvertebrate size and indicator value are linearly correlated, resulting in a non-linear increase in organism densities with respect to size. Size differences were converted to differences in relative abundance by assuming an allometric size (S) density (d) relationship (S = d^-0.75^; [45]) and taking organism size as the diameter of a circle (which was mapped in two-dimensions). The seven classification groups of macroinvertebrate size described by Tachet et al. (2000) [46] are provided for comparison.

	Hypothetical, Size-biased Indicator System (I_Hs_)									
Indicator score	1	2	3	4	5	6	7	8	9	10
Organism size (mm)	2.5	3.75	5.63	8.44	12.66	18.98	28.48	42.71	64.41	96.27
Relative density (per unit habitable space)	130.04	75.73	44.11	25.69	14.96	8.71	5.07	2.96	1.71	1
	Organism size classes from the trait database of Tachet et al. (2000)									
Size classes	1	2		3	4		5	6		7
Organism size (mm)	<2.5	2.5–5.0		5.0–10.0	10.0–20.0		20.0–40.0	40.0–80.0		>80

The size vs tolerance score of diatom indicator systems was assessed by Spearman’s rank correlation, applying Rimet & Bouchez’s [47] biovolume classes; omitting indicators that were not included in their summarized database. The lack of data on biovolume precluded statistical analysis for macroinvertebrates and marine benthic organisms.

### Misclassification

The influence of misclassified taxa can be defined by: (i) their proportionate occurrence, and (ii) their degree of misclassification. Scenario (i) was addressed by considering an indicator, misclassified by 3 ranks below the mode that was sampled with a modal frequency and represented an increasingly large proportion of the modal abundance (0–67% of the mode, cf. 0–18% of the total sample). Scenario (ii) was addressed by including an indicator representing 20% of the modal population which was misclassified with the lowest score (one) in simulations considering an increasingly distant mode (ranging from 3–10).

Although Chironomidae and Oligochaeta are classified as the most pollution tolerant indicators in the BMWP (scoring two and one, respectively; cf. IBMWP, MCI, pan-US) they occur in habitats of all qualities [48,49]. Based on the averaged percent-abundance for 29 contrasting rivers (the UK’s ECN long-term monitoring program [39]) where Chironomidae and Oligochaeta represented over one-quarter of macroinvertebrate taxa (mean±sd: 15.1±8.9 and 11.1±17.3, respectively), the influence of their misclassification was evaluated by assigning Chironomids and Oligochaeta 25% of the total abundance (12.5% each) in simulated runs where the mode spanned the range of BMWP scores (1–10). A more comprehensive evaluation of misclassified BMWP taxa was based on the revised indicator scores presented by Walley & Hawkes [32] where it was reported that three quarters of BMWP taxa were misclassified. Here, the probability of selection was defined by the revised BMWP scores and index values were subsequently calculated from both the original and revised tolerance scores, comparing the absolute difference in their derived index values.

### Composite, net bias

As the effects of respective biases are additive, I combined the biases of truncated frequencies, skewed indicator distribution and misclassified taxa for the BMWP (other biases cannot realistically be assumed for real data) to describe the trend in net index bias across the range of BMWP scores by comparison with the hypothetical system, I_H_. The resultant predicted generalization was subsequently tested by comparison with data from 309 sites on Scottish rivers [40], representing a range of environmental qualities.

## Results

### Truncated frequencies

**Fig 3:**
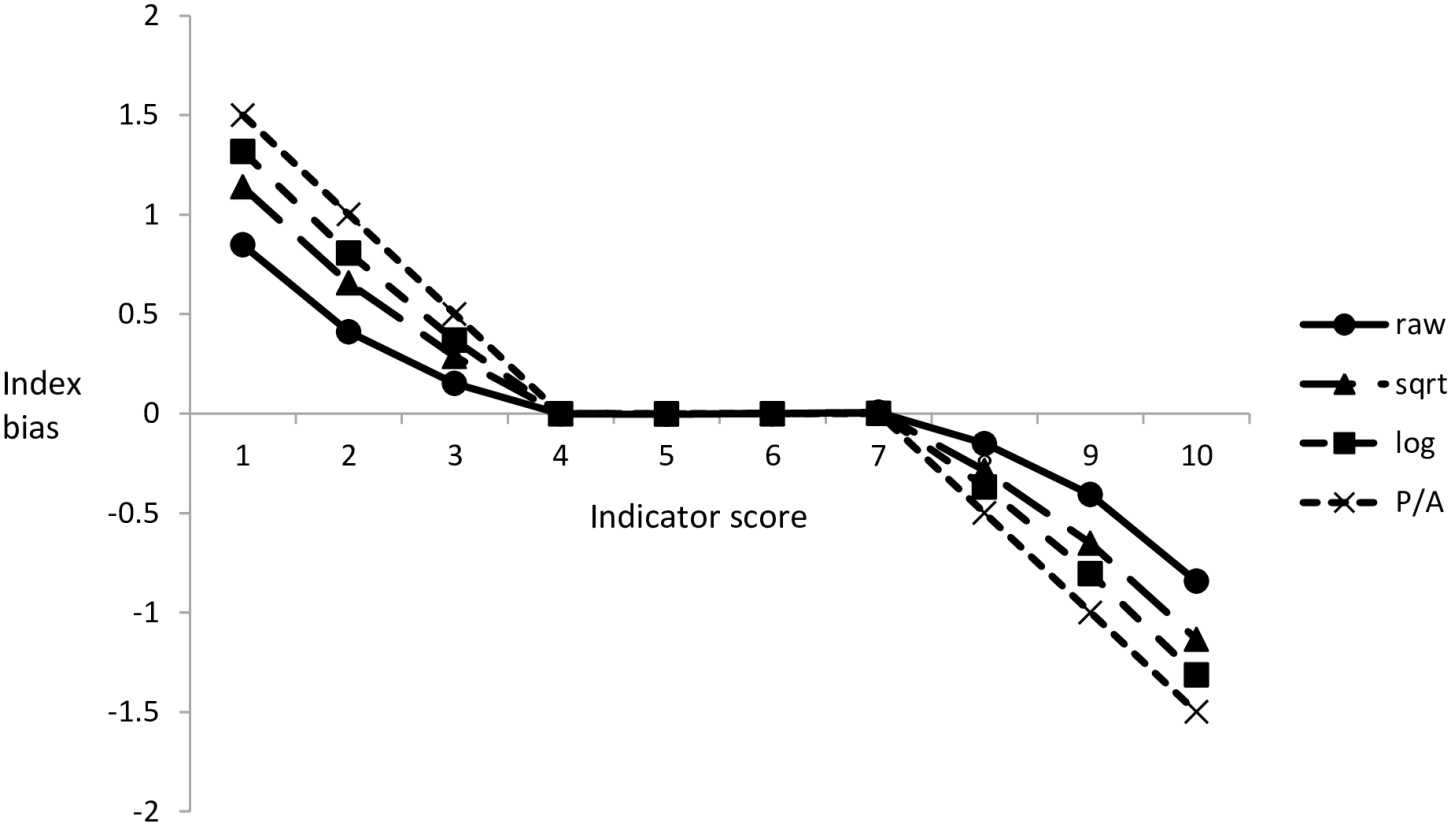
Truncated frequency distributions at the extremes of the range of indicator scores causes a positive (lower end) and negative (upper end) bias in the derived value for an idealized biotic index, resulting in an overall compression of the index range.

The truncation of the frequency distributions caused a positive and negative bias for the lowest and highest index scores, respectively (Fig 3). Bias was greatest for presence/absence data and lowest for non-transformed data with a range compression represented by: presence/absence (7.00) < logarithmic (7.37) < square-root (7.72) < raw (8.30).

### Indicator evenness

**Fig 4:**
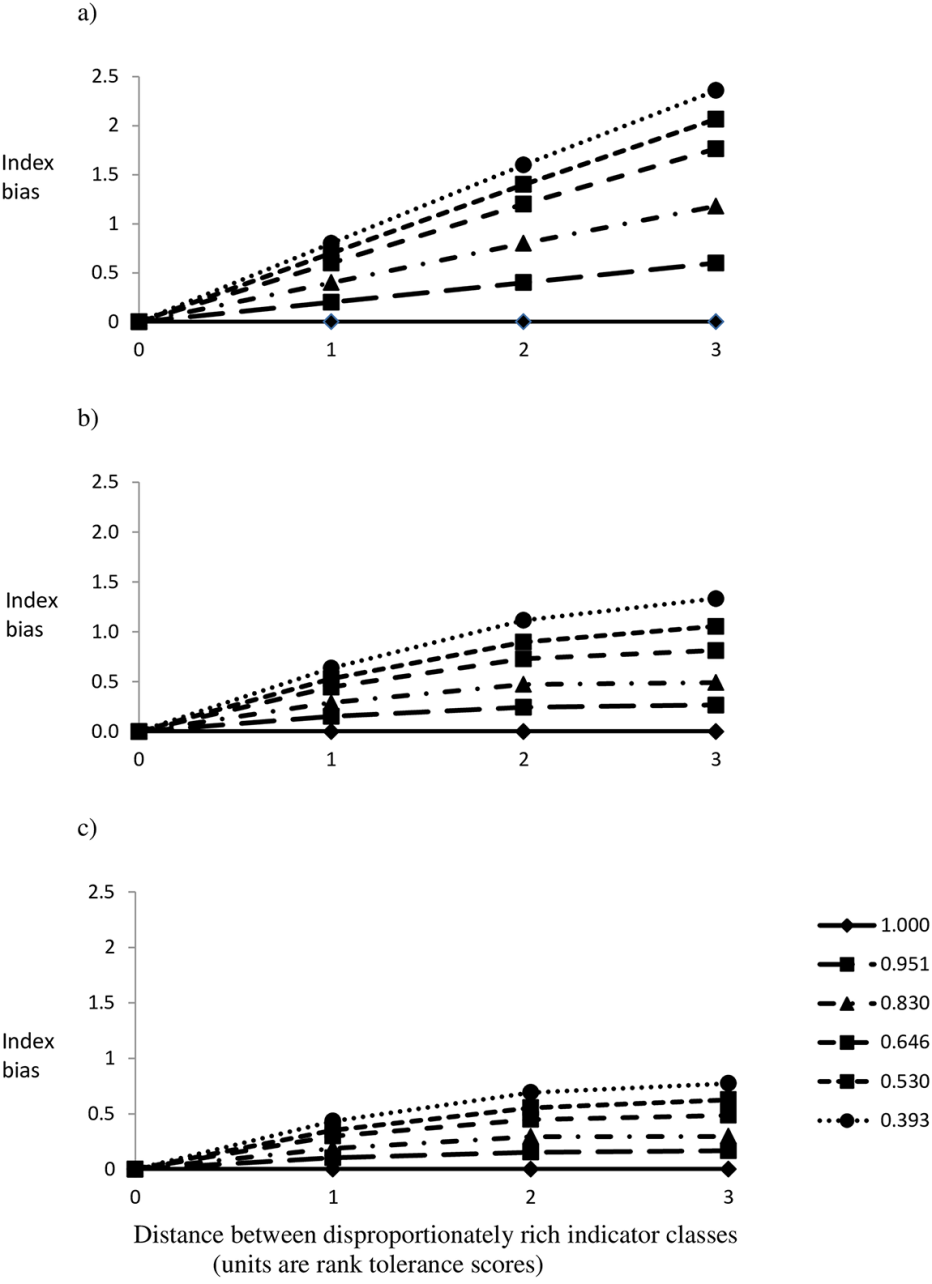
Results from simulation models comparing contrasting evenness (Simpson’s D, inset) and resultant bias (y-axis) as the distance of the skewed class to mode ranged over 1, 2 and 3 rank classes (x-axis) for (a) presence/absence, (b) log transformed, (c) square-root transformed.

Skewing indicator distributions in I_H_ caused a bias in the direction of the taxonomically rich indicator class. Divergence increased with the severity of data transformation and as the disproportionately rich class became more distant from the mode (Fig 4a–4c). Indices based on raw abundance data always conformed to the mode.

The evenness of indicator distributions differed considerably between the reviewed indicator systems, with none completely equitable (Table 1). Overall evenness was a good indicator of skew amongst the lower scoring classes in respective indicator systems (Pearson’s correlation 0.444, p = 0.04), which tended to be more extreme among low-value (pollution tolerant) indicators compared to high-value indicators (Table 1).

**Fig 5:**
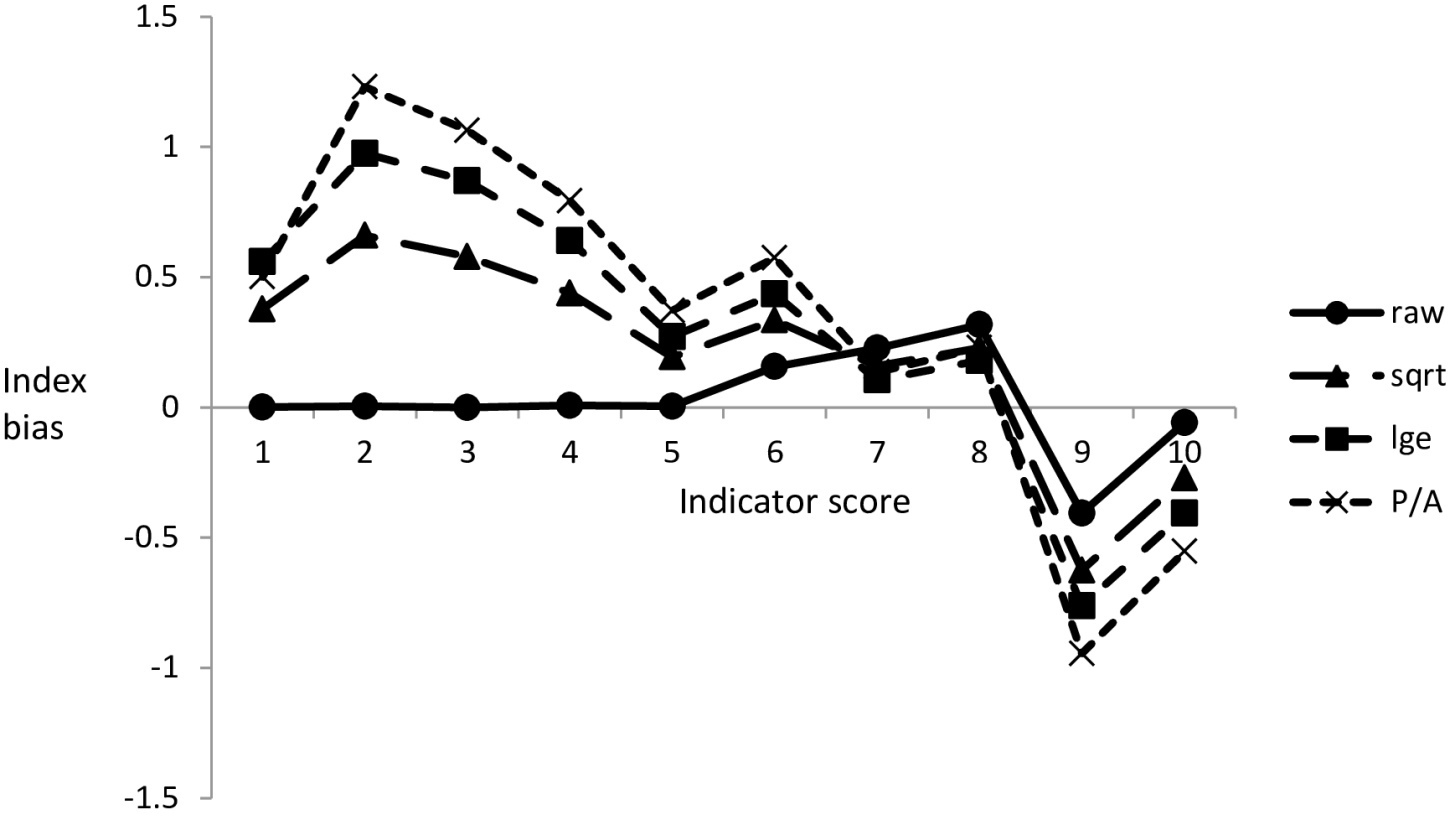
Simulation results illustrating the interaction between skewed indicator richness and the statistical treatment of abundance data (see inset) along the scoring range of the BMWP.

BMWP-based indices revealed a positive deviation for low values and a negative deviation for high values for indices based on transformed abundance data (Fig 5). The raw abundance index was unbiased for low scores and alternately positively then negatively distorted for scores above five due to the distorted frequency distributions associated with the null group (indicator rank = 9).

### Size bias

**Fig 6:**
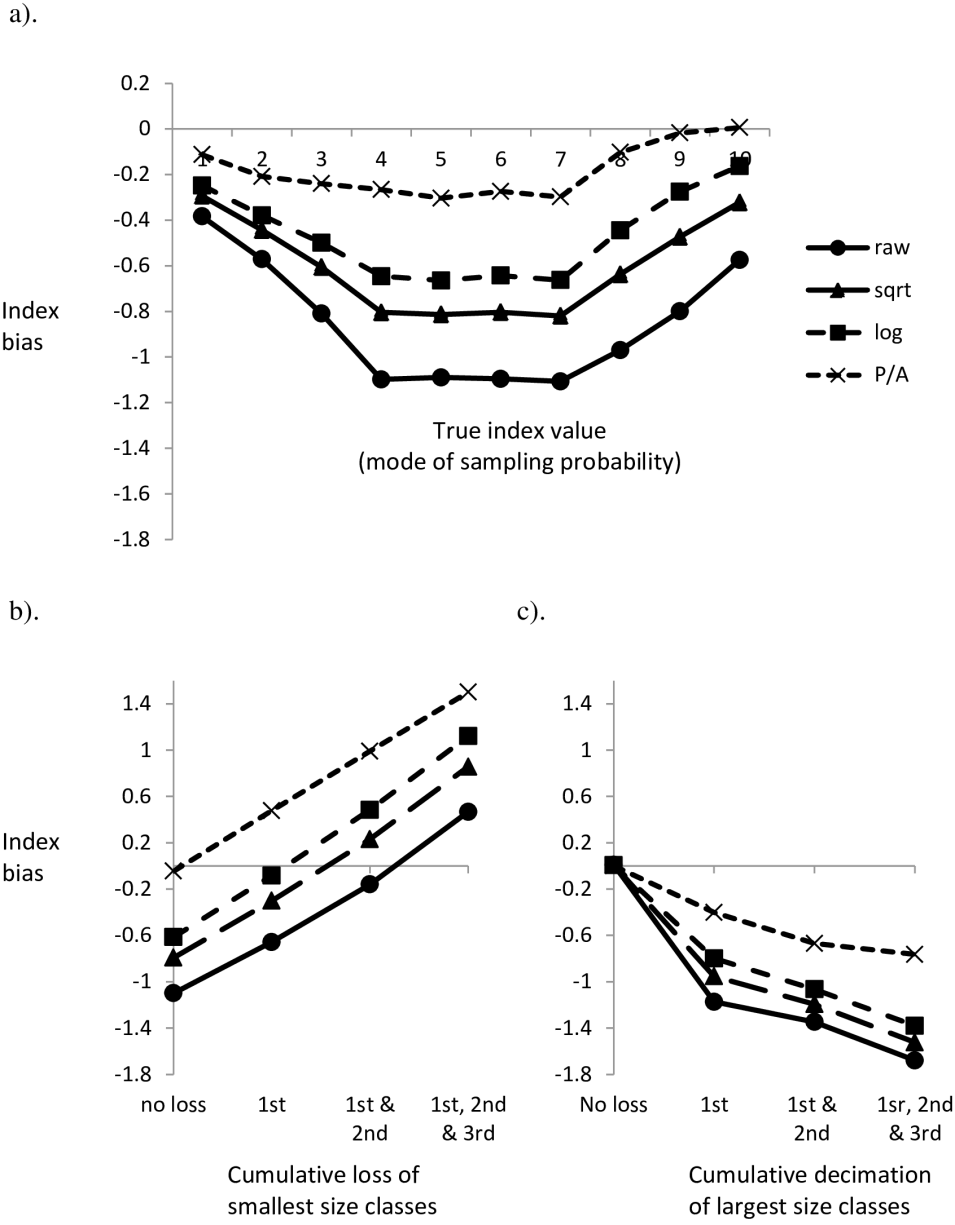
A perfect correlation between indicator size and indicator score causes a systematic bias in index values that varies with the treatment of abundance data (see inset). (a) overall negative bias over the range of indicator scores: (b) antagonistic interaction associated with the failure to collect the smallest three size-indicator classes (indicator mode = 4); (c) synergistic interaction associated with the decimation of the three largest size-indicator classes (indicator mode = 7).

The systematic correlation between size and indicator scores resulted in an overall negative bias that was reduced by data transformation (Fig 6a). Despite detrending for index compression, size bias was associated with a marked “end-effect” as the influence of size was mitigated by the truncated frequency distributions (Fig 6a). Both random colonization and the preferential colonization of vacated space by small-sized organisms was associated with a negligible difference in index scores (data not presented). Omission of the smallest organisms increased the derived index value (Fig 6b), compensating the overall size-abundance-indicator bias (Fig 6a & 6b). Combining correlated size—tolerance scores with the systematic loss of larger organisms resulted in a negative bias that increased as the number of large-sized indicator classes affected increased; again, overall bias was mitigated by the increasingly harsh data transformations (Fig 6c).

Rimet & Bouchez’s [46] biovolume classes indicated a negative correlation between diatom size and TDI tolerance scores (-0.208, p<0.001), however, other indicator systems demonstrated weak positive correlations with size: CEE (0.173, p<0.01), IPS (0.084, p<0.05), Van Dam pH (0.177; p<0.001). Despite the absence of comprehensive data for freshwater macroinvertebrates it was notable that the comparatively small Chironomidae and Oligochaeta often represented the most tolerant scoring classes (e.g. BMWP, IBMWP, MCI, pan-U.S.).

### Misclassification

**Fig 7:**
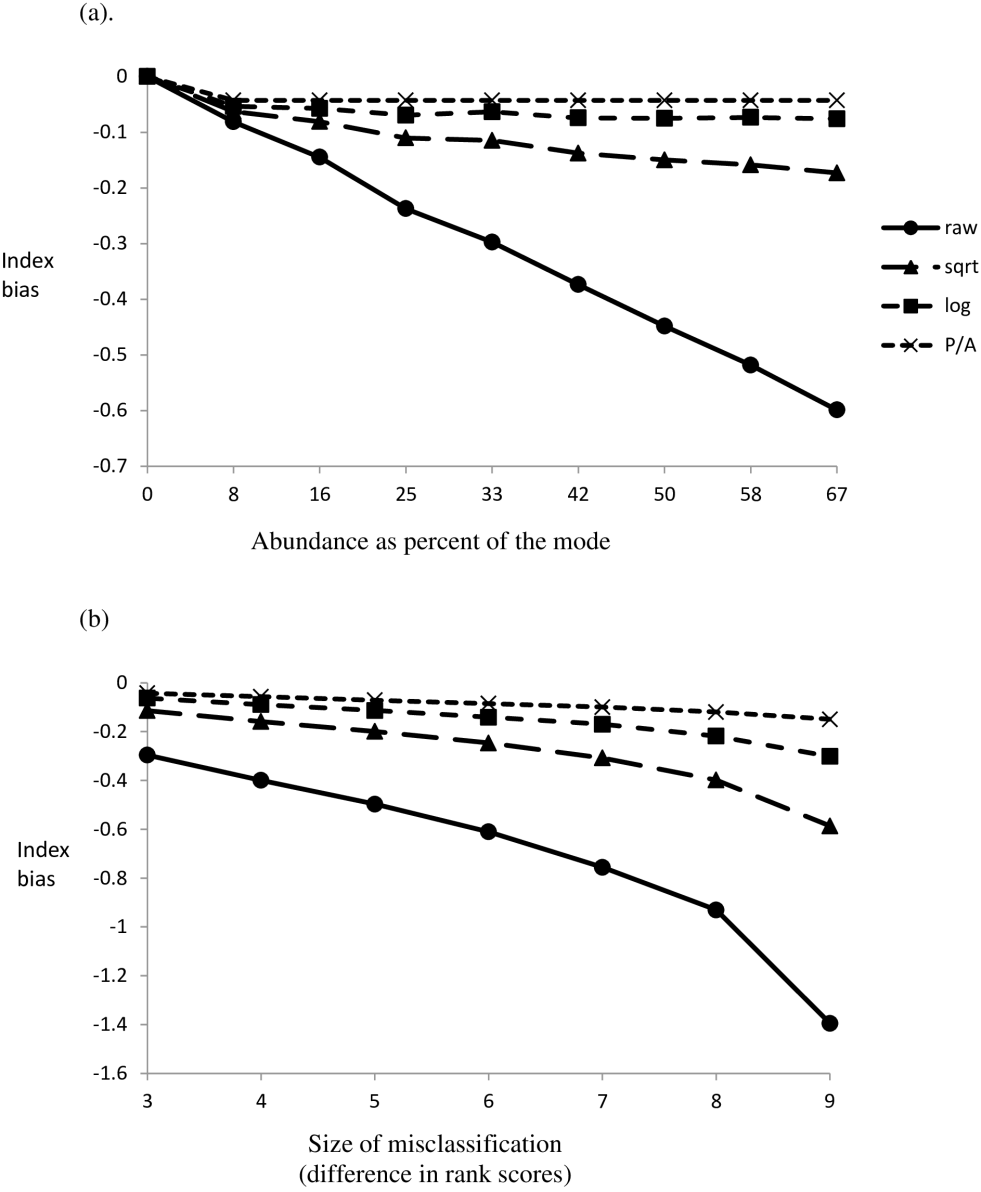
The treatment of abundance data (see inset) influences the degree of bias associated with two scenarios of misclassification, illustrated by (a) a single taxa misclassified by 3 rank values as its proportionate contribution to the mode is increased. (b) The presence of a single mis-classified indicator representing 20% of the mode as the degree of misclassification increases from 3 to 9 rank scores.

The effect of misclassifying a single taxon by three scoring classes was relatively small and decreased with the severity of data transformation (Fig 7a and 7b). Increasing the margin of misclassification increased bias (Fig 7b).

**Fig 8:**
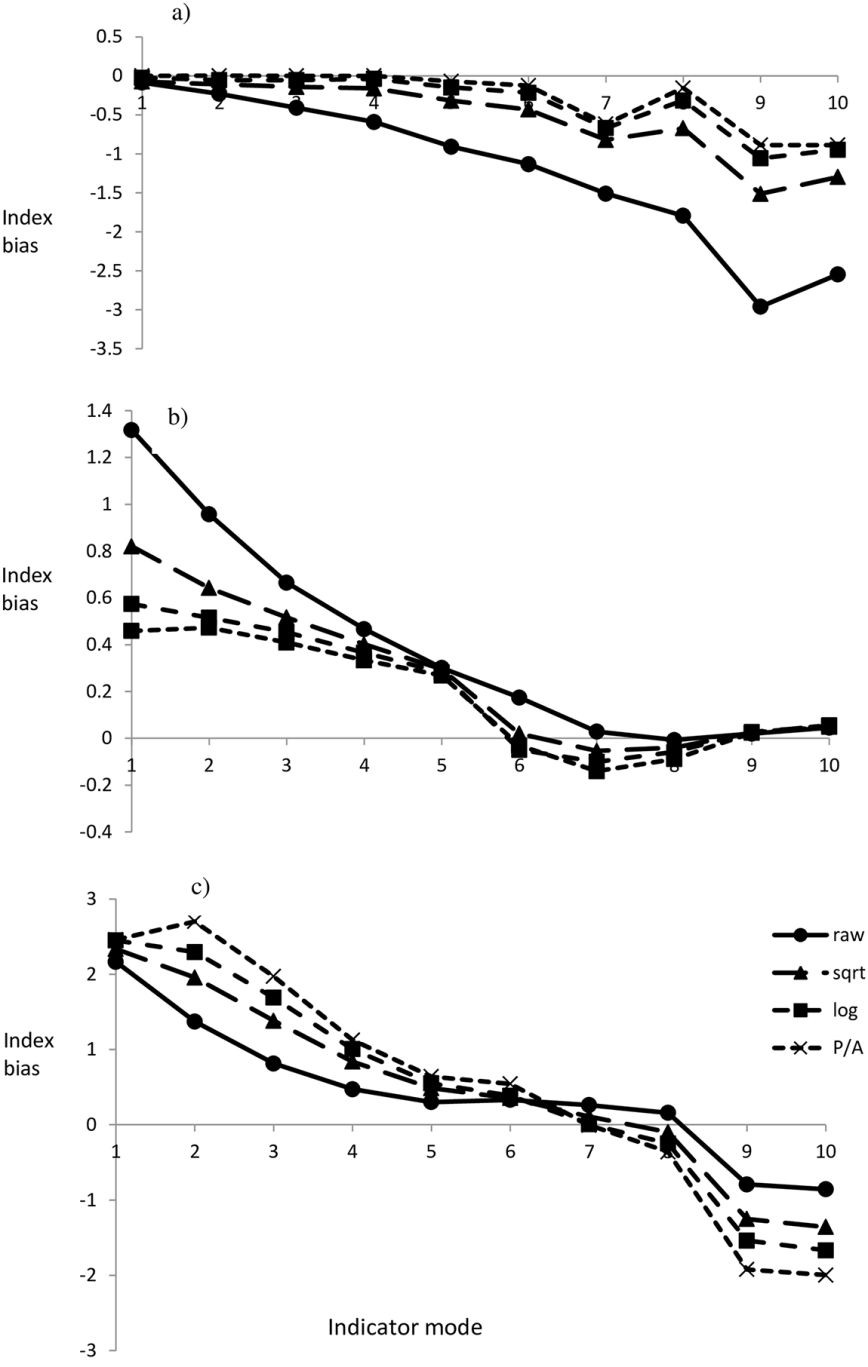
Bias (y-axis) in the derived index value associated with increasingly complex scenarios in BMWP-based indices as the mode ranges from 1 to 10 (x-axis), reflecting. (a) the misclassification of Chironomidae and Oligochaeta when they contribute 25% of individuals; (b) multiple misclassified taxa defined by Walley and Hawkes [32]; (c) Net bias associated with range compression, taxonomic skew and misclassified taxa.

For the BMWP-based indices, bias associated with misclassification of Chironomidae and Oligochaeta increased as the modal value increased and was consistently greater for mildly transformed and non-transformed data (Fig 8a). The net effect of all misclassified taxa (according to [32]) was most pronounced for raw abundance data where it accounted for a positive bias of 1.3 units (Fig 8b). In general the risk of bias decreased with increasing index scores and became negative for transformed data between index values 6 to 9 (Fig 8b).

### Composite, net bias

For BMWP-based indices the additive effect of range compression, skewed indicator distribution and misclassifications described a trend of a gradually decreasing positive bias across the low-scoring range (1–6), switching to a negative bias for high-scoring values (9–10; Fig 8c). In general the bias tended to be greater as the severity of data transformation increased (Fig 8c).

**Fig 9:**
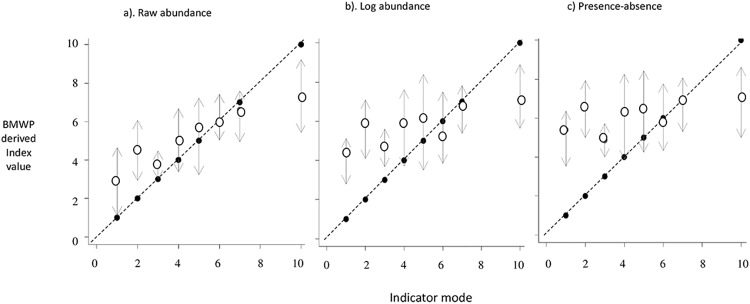
Bias in derived BMWP-based index values compared to the indicator mode for 309 rivers of variable quality throughout Scotland considering contrasting treatments of abundance data. a) raw abundance, b) log-transformed, c) presence-absence. Solid circles represent the indicator mode, open circles represent the derived index value, arrows indicate the range. (N.B. no sites were characterized by a mode of eight, nine is a null group).

Net bias was evident in the derived index values for the 309 Scottish rivers, broadly corresponded to the predictions of the simulated analysis (Fig 8c vs Fig 9a,9b and 9c). The contraction of the range increased with the severity of data transformation: raw (8.09) > square-root (6.87) > log (6.1) > presence/absence (5.09). Deviations were consistently lower than the mode for low-scoring values (1–5), and increased with the severity of data transformation, whereas deviations were negative at the highest modal value (with the difference between respective data treatments less distinct; Fig 9).

## Discussion

Despite the considerable scope to compensate for bias, the interdependence of sampling equipment, laboratory processing and data treatment limit the refinement of index accuracy to a strategy of optimal compromise. Special attention should be given to the risk of positive bias associated with low index values. For the BMWP indicator system this is primarily associated with the depauperate richness of low-scoring indicators and the potential disproportionate efficacy in the collection of small-bodied (low scoring) organisms. These issues are common to many of the assessment methods detailed in Table 1. Context of application provides an appreciation of the risk to biodiversity management. In the UK quality classification is based on an Observed/Expected ratio (test v reference assemblages; [38]). Given that the BMWP index for presence-absence data can be as low as 3.08 at a reference site (or 4.31 for a reference “type”; N = 12; 3-season samples [38]) and that low-score positive bias can exceed 100% of the true index value, naturally low-scoring sites may need to be all but devoid of life to fail quality standards. This specific example is contextualized by the observation that most biotic indices present size bias and skewed indicator distributions that are generally comparable and sometimes more extreme than the BMWP (Table 1).

### Taxon richness evenness

Numerous options could be exploited to develop indicator systems that are more equitable in terms of indicator size and richness. Representing 84 indicator “families” (excluding Oligochaeta) the BMWP exploits less than half the 210 families of UK macroinvertebrates [50]. Similarly, the widely used FBI is limited to aquatic insects, excluding Crustaceans, Annelids and Molluscs. Increasing the taxonomic resolution of indicator assignment provides an alternative option. Comparing Hilsenhoff’s family-level FBI with his species level BI demonstrates how higher taxonomic resolution can deliver greater equitability (Table 1). Ultimately the scope to adjust indicator equitability is limited by nature. Biogeographic phenomena can give rise to a particularly challenging evaluation of bias when the regional species assemblage represents a skewed fraction of the designated indicator taxa [41]. Under these scenarios, reviewing indicators’ traits (dispersal, life-cycle, etc.) could help distinguish between potential colonists and taxa that are otherwise associated with a biogeographically restricted distribution.

### Universal indicators

Issues of biogeography have been brought to the fore by efforts to harmonize assessment methods across political frontiers. While the elaboration of pan-continental indicator systems is an enticing idea, the regional specificity of indicator systems is, to a large degree, grounded in the differential sensitivity of organisms. Describing a pan-European indicator system for diatoms, Besse-Lototskaya *et al*. [29] essentially averaged the indicator scores from seven different indicator systems. This strategic compromise required the creation of “intermediate” ranks that merge indicators previously assigned different indicator scores [51]. As a result, the total number of classes was increased and the subsequent indicator distribution is highly inequitable (Table 1). Tackling the issue via empirical analysis, Carlisle *et al*. [30] derived macroinvertebrate indicators for the US in relation to various water quality characteristics by applying a combination of ordination (to describe quality gradients) and weighted-averaging (to derive indicator scores), resulting in pleasingly symmetrical indicator distributions (Table 1). However, both these approaches represent an increased risk of error. Essentially brushing over the regional differences in sensitivities, these biogeographic compromises imply that at any given location the probability of misclassification is increased [51].

### Taxonomic skew and indicator abundance

The treatment of abundance data provides considerable scope to off-set bias. Raw abundance data can mitigate the effects of uneven indicator richness. Conversely, transformation of abundance data can be used to give more emphasis to indicator richness and otherwise dampen unrepresentatively high abundance data. Some of the key issues of differential richness can be identified by descriptive analysis. For example, synchrony in the life-cycles of Ephemeroptera, Plecoptera and Trichoptera (EPT) may synergistically interact with a skew in their indicator distribution and cause temporal instability in biotic indices, a phenomena that tends to be more extreme where seasonal differences are more pronounced [52, 53]. Considering the ECN data, the BMWP-based indices were significantly higher in spring at 12 sites based on presence-absence data, compared to 5 sites based on raw abundance data, highlighting the damping effect of raw-abundance data on the springtime peak in EPT richness. The interaction between indicator skew and abundance data are also revealed by differences in the index range. For the ECN data, the BMWP-based index value ranged from 2.40–8.11 (presence-absence) compared to 1.02–9.37 (raw abundance) across sites, representing an increase of 64% in the overall range. However, the extent to which the treatment of abundance data can be used to improve accuracy must also consider other aspects of assessment design.

The theoretical relationship between organism size, abundance and disturbance led Warwick [26] to propose a method of bioassessment defined in terms of the ratio of organism size and abundance. The trend in indicator size and indicator scores in some of the indicator systems described in this study appears to provide qualified support for the premise that smaller organism are often comparatively tolerant to environmental degradation (albeit a generalization that is subject to many exceptions). The highly exaggerated size-bias of simulated models illustrated a scenario that is only vaguely approximated in some cases for real indicator systems. Yet, as the absence of a correlation merely confirms that there is no *systematic* size bias, this provides little room for reassurance. Any size difference can be associated with bias whenever two or more co-existing organisms differ in both size and indicator class. Anecdotally, it is worth contemplating the extreme size difference between the largest macroinvertebrate of the BMWP system, Astacidae (120 mm; BMWP = 8) and the some of the smallest, Chironomidae and Oligochaeta (<5 mm; BMWP = 2 and 1, respectively). Evidently, an average kick-sample is likely to capture rather more Chironomidae and Oligochaeta than crayfish. Similar size—tolerance score disparities are apparent in other indicator systems (e.g. IBMWP).

The pernicious effects of organism size has been more commonly addressed by researchers working with microscopic organisms, presumably because differences in organism size can be more extreme and often more tractable for these groups. Some Saprobic indicator systems incorporate five orders of magnitude [20] diatoms range over three (5–2,000μm; [54]), while macroinvertebrates typically spans less than two (2.5 mm– 80.0 mm; [46]). Several Saprobic and diatom indices incorporate abundance data via categorical classes, using this as an adjustment system to compensate for differences in size whereby fewer larger-bodied individuals are required to achieve the equivalent classification of “high abundance” compared to smaller individuals [20, 55]. As diatoms are encased in a siliceous cell, organism size is essentially constant and facilitates size-based generalizations [46, 50]. Estimates of total biovolume can therefore be derived by multiplying cell abundance by the species-specific biovolume.

Warwick et al. [14] explored various options to down-weight abundant indicators via statistical transformation prior to the derivation of the AMBI, concluding with a recommendation to apply the “moderate” adjustment of square-root transformation. Taylor’s Law [21] represents an empirical model that characterizes the abundance distribution of populations and otherwise identifies optimum statistical transformations. Applying the theoretical imperative of Taylor’s Law to log-transform macroinvertebrate abundance prior to index derivation demonstrated a significant increase in the precision and accuracy of a broad range of bioassessment metrics [56]. Similar aggregated distributions for a wide range of other organisms [21, 57] suggests that similar improvement might be achieved with other indicator taxa.

The systematic avoidance of small organisms represents an extreme scenario of size bias that can be particularly acute for low-scoring indicators. The risk of size-avoidance is indicated by considering body-size in relation to size-selective survey methods. For protocols employing nets and sieves, it is primarily defined by mesh size. Amongst aquatic macroinvertebrates attention has necessarily focused on the low-scoring Diptera and Oligochaeta with body morphologies that approximate narrow cylinders. In many protocols the mesh-size of a typical net is around 500–600 μm and sometimes as large as 1mm [35, 58]. As most final instar Chironomidae have a head capsule width < 350μm [59] and the body-width of aquatic Oligochaeta is often < 400 μm [49] these taxa are presumably systematically underrepresented by bioassessment sampling methods. Ironically their underrepresentation in samples may represent a fortuitous correction for taxa whose indicator values are often grossly misclassified.

### Misclassification

Taxa are misclassified for a variety of reasons including the methods used to derive indicator scores, obliged pragmatism, and insufficient knowledge. Pragmatism is important in the assignment of indicator values at course levels of taxonomic resolution when the indicator values of constituent taxa are known to differ [33]. It is exemplified by the frequently lamented misclassification of Oligochaeta and Chironomidae that are generally assigned low scores (e.g. IBMWP, FBI, SIGNAL). Distinguished as the most tolerant BMWP indicator, Oligochaeta occur in habitats of good and bad quality [49]. Chironomids, represented by more than 10,000 species worldwide, are similarly present in almost all freshwater habitats [48]. Compromise to their environmental ubiquity is illustrated by the course-resolution FBI, where chironomids are assigned to two classes (distinguished as “Blood-red Chironomidae (Chironomini) 8, other (including pink) Chironomidae 6”) compared to the high-resolution BI where their diverse genera occupy the entire range of 11 tolerance classes [16, 60].

Historical precedent can represent an important nuance for indicators of general environmental quality as management focus changes from point source, organic inputs to more holistic definitions of pollution. If the definition of environmental quality changes, the relevance of previously established quality indicators may be compromised. Identifying potential causes of misclassification can be particularly problematic when indicators have been assigned by the occult art of expert opinion [61], where the criteria of indicator assignment and the gradient of ranked scores are rarely explained. Precise meaning is also obscured when indicators are assigned via *a posteriori* methods of ordination [30]; based on the statistical comparison of multi-species assemblages, the derived indicator scores for individual taxa are implicitly dependent on the abundance distributions of all other taxa. The more common method of iterative weighted-averaging in relation to an *a priori* quality gradient to derive “ecological optima” (after [8]; e.g. [60]), provides a simpler statistical definition of tolerance scores. Although assumption about unimodal distributions, competitive displacement and data gaps (zero occurrences) can be problematic [42], this individualistic analytical perspective offers a more parsimonious model for indicator assignment. However, the opportunity to reduce the spatio-temporal “noise” of abundance data and generate more representative weighted-averages via data transformation [56] appears to have been largely overlooked in the derivation of indicator scores.

Recognizing that their scientific objectives were fundamentally determined by data availability, the pioneers of biotic indices counselled the revising of indicator systems as more data became available [33, 60]. Analyzing a dataset of 1700 samples, Wally & Hawkes [32] found that three quarters of BMWP taxa were misclassified with almost twice as many representing inappropriately high (44%) as opposed to inappropriately low (24%) scoring ranks. Considered in the wider context of this review, it is worth noting that their re-evaluation also resulted in a more equitable distribution of indicators [32].

The revolution in data acquisition delivered by next generation DNA sequencing offers an exciting opportunity to “re-boot” methods of bioassessment [62, 63]. The capacity to bulk process homogenized benthic samples [62] and indirectly detect organisms from water samples as “environmental DNA” (e-DNA; via faeces, urine, cell/tissue fragments, etc. [64]) enables a rethink on sample collection and offers the possibility to address some of the problematic issues associated with net mesh-size and morphological taxonomy. Barcoding provides a quick turnaround on high-resolution data from benthic samples that can include immature specimens and groups that are otherwise taxonomically challenging (e.g. Diptera, Oligocheata). As such, it could be used to develop more comprehensive indicator systems and help reduce bias associated with body-size and the skewed richness of contrasting indicator classes. However, the application of this new technology brings its own risks of bias to the derivation of biological quality. In aquatic ecosystems e-DNA can persist for extended periods (days to weeks [65]), creating potential difficulties for site specific monitoring that could be particularly acute for rivers and coastal waters [64]. As e-DNA is ubiquitous it is present in the benthos and may therefore represent contamination in homogenized benthic samples. Laboratory procedure is also a critical issue for bar-code bioassessment: primers can fail to pick up the DNA of some organisms while the DNA of others can be amplified to different extents and confound quantitative comparison [62, 63, 66]. Nonetheless, the increasing investment in DNA barcoding [62, 67] suggests that the design and application of bioassessment might need to adapt to the pros and cons of this new technology and its associated caveats for the interpretation of biotic indices.

In the absence of an explicit reference condition, any metric of ecological quality has limited meaning because the expected value (for the non-degraded system) is unknown. Expressing a biotic index in the context of the reference condition summarizes the relative quality of ecological conditions (the resultant ratio is often referred to as an “Ecological Quality Ratio”, EQR [3]). Expressing biotic indices as an EQR also provides a precaution against the risk of systematic bias that has been considered in this study. Assuming the reference condition is accurately assigned, the consequent effect of bias can be inferred from knowledge of ecological similarity between replicate samples [68]. As individual biases are additive, their net effect is expected to result in a normal distribution of errors around the average net bias [69]. If survey protocols are standardized, this error variance is defined by the sum of biases associated with sample variability. As the risk of bias in the test sample and reference sample are the same, their overall respective biases will—on average—cancel. However, for any particular comparison, residual differences in net bias will be present and can be estimated in terms of the overall error variance for reference comparisons. This emphasizes the importance of standardized survey protocols, accurate reference assignment and sample replication in the derivation of the comparative ratio.

### Conclusion

This study has demonstrated the risk of bias associated with a wide range of biotic indices, providing a detailed example based on the original BMWP indicator system. Assessment was facilitated by the comprehensive data available for review. To the pioneers of bioassessment [33, 60], access to such data was considered essential to progress.

Preceded by a long history, bioassessment has only recently begun to gain recognition from environmental managers [1, 7]. The severity of contemporary global change presents a particularly challenging agenda. Simple metrics of biodiversity provide an inadequate summary of ecological degradation [5] and highlight the need for metrics that can provide information on specific aspects of biological quality. Given the prominence of biotic indices in national monitoring they are arguably the single most influential metric defining the ecological management of aquatic resources. This emphasizes the need to maximize the accuracy of biotic indices and to clearly communicate the information provided by their summarized numerical value. Reporting biotic indices as a comparative ratio with an appropriate reference enables the quantification of net bias and the consequent reliability of the index-ratio to be estimated. The effects of body-size, abundance, richness and ascribed indicator scores provide four reasons why end-users should check the estimated accuracy whenever quality ratios have been derived from a biotic index.

## Supporting Information

S1 File Biotic Indices References. (DOCX)
