# Supplementary material for: Four Reasons to Question the Accuracy of a Biotic Index; the Risk of Metric Bias and the Scope to Improve Accuracy
Source: PLoS One. 2016 Jul 8;11(7):e0158383. doi: 10.1371/journal.pone.0158383 (PMC4938548; doi:10.1371/journal.pone.0158383)
Supplement: S1 File — (DOCX) [file pone.0158383.s001.docx]

**Biotic Indices References**

- **Re: Table 1; Four reasons to question the accuracy of a biotic index; the risk of metric bias and the scope to improve accuracy.**

Alba-Tercedor J. & Pujante A.M. (2000) Running-water biomonitoring in Spain: opportunities for a predictive approach. pp. 207-216, In: *Assessing the biological quality of freshwaters; RIVPACS and other techniques* Wright J.F., Sutcliffe D.W. Furse M.T. (editors). Freshwater Biological Association, Ambleside.

Armitage P.D., Moss D., Wright J.F. et al. (1983) The performance of a new biological water quality system based on macroinvertebrates over a wide range of polluted running-water sites. *Water Research,* 17: 333-347.

Besse-Lototskaya, A., Verdonschot, P. F. M., Coste, M., & Van de Vijver, B. (2011). Evaluation of European diatom trophic indices. *Ecological Indicators*, *11*(2), 456–467.

Borja, Á., Franco, J., Pérez, V., 2000. A marine biotic index to establish the ecological

quality of soft bottom benthos within European estuarine and coastal

environments. Marine Pollution Bulletin 40, 1100–1114.

Carlisle, D. M., Meador, M. R., Moulton, S. R., & Ruhl, P. M. (2007). Estimation and application of indicator values for common macroinvertebrate genera and families of the United States. *Ecological Indicators*, *7*(1), 22–33.

Cemagref, (1982) Etude des methods biologiques quantitatives d’appréciation de la qualité des eaux. Rapport Division Qualité des Eaux Lyon – Agence de l’Eau Rhône – Méditerranée – Corse, Pierre – Bénite, 28 pp.

Chessman, B. C. (2003). New sensitivity grades for Australian river macroinvertebrates, (1998), 95–103.

Damuth, J. (1981) Population density and body size in mammals. Nature. 290, 699-700.

Davy-Bowker J., Murphy J.F., Rutt G.R., Steel J.E.C. & Furse M.T. (2005) The development and testing of a macroinvertebrate biotic index for detecting the impact of acidity on streams. *Archiv Fur Hydrobiologie.* 163, 383-403

Descy J.P. (1979) A new approach to water quality estimation using diatoms. *Nova Hedwigia* 64, 305-323

Extence, C. A., Balbi, D. M., & Chadd, R. P. (1999). River flow indexing using British benthic macroinvertebrates: a framework for setting hydroecological objectives, *574*, 543–574.

Hilsenhoff W.L. (1987) An Improved Biotic Index of Organic Stream Pollution. The Great Lakes Entomologist, 20, 31-40

Hilsenhoff, W. L. (1988). Rapid field assessment of organic pollution with a family-level biotic index. *Journal of the North American Benthological Society*, 65–68.

Kelly M.C. (1998) Use of the Trophic Diatom Index to monitor eutrophication in rivers. *Waters Research,* 32, 236-242.

.Leonardsson K, Blomqvist M. & Rosenberg R. (2009) Theoretical and practival aspects on benthic quality assessment according to the EU Water Framework Directive – examples from Swedish waters. *Marine Pollution Bulletin*, 58, 1286-1296.

Mistri M. & Munari C. (2008) BITS: a SMART indicator for soft-bottom, non-tidal lagoons. *Marine Pollution Bulletin*, 56, 587-599

Simboura, N. & Zenetos, A., 2002. Benthic indicators to use in ecological quality classification of Mediterranean soft bottom marine ecosystems, including a new biotic index. *Mediterranean Marine Science*, 3 (2): 77-111.

Sládeček, V. (1986) Diatoms as indicators of organic pollution. *Acta Hydrochim. Hydrobiol.* 14, (5), 555-566.

Stark J.D. & Maxted J.R. (2007) A biotic index for New Zealand’s soft-bottomed streams, *New Zealand Journal of Marine and Freshwater Research,* 41, 43-61

Tachet, H., Richoux, P., Bournard, M. & Usseglio-Polatera, P. (2000) Invertebres

d'eau douce. Systematique, biologie, ecologie. CNRS Editions.

Watanabe T., Asai K & Houki A. (1986) Numerical estimation of organic pollution of flowing water by using epilithic diatom assemblage – diatom assemblage index (DAIpo). *The Science of the Total Environment.*

Ziemann, H., 1971. Die Wirkung des Salzgehaltes auf die Diatomeenflora als Grundlage für eine biologische Analyse und Klassifikation der Binnengewässer. Limnologica 8, 505–525.
